# Auswirkungen des Klimawandels auf die Gesundheit und Rolle des Gesundheitssektors bei der Reduktion von Treibhausgasemissionen

**DOI:** 10.1007/s00120-025-02699-y

**Published:** 2025-10-10

**Authors:** E. Rothwell, Jonny Groome

**Affiliations:** 1 University Hospitals Bristol and Weston NHS Trust, Bristol, Großbritannien; 2 Paediatric Anaesthetic Department Office, Level 4, The Royal London Hospital, Whitechapel Road, E11FR London, Großbritannien

**Keywords:** Klimakrise, Planetare Gesundheit, Nachhaltigkeit, Nachhaltige Krankenhäuser, Klimaschutz, Climate crisis, Planetary health, Sustainability, Sustainable hospitals, Climate mitigation

## Abstract

Die Klimakrise gilt als größte Bedrohung für die menschliche Gesundheit, und paradoxerweise ist der Gesundheitssektor für 5 % der weltweiten Treibhausgasemissionen verantwortlich, die diese Krise befeuern. Die Emissionen sind größtenteils bedingt durch Einrichtungen mit hohem Treibhausgasausstoß, durch Energieverbrauch, komplexe globale Lieferketten, Transport und Arzneimittel. Angesichts seiner Funktion als Garant der Gesundheit heutiger wie auch kommender Generationen muss der Gesundheitssektor Maßnahmen ergreifen, um seine negativen Auswirkungen auf die Umwelt zu minimieren. Zu den emissionsreduzierenden Strategien zählen eine nachhaltige Infrastruktur, Innovationen in der klinischen Praxis sowie Verbesserungen in Bezug auf Beschaffung und Lieferketten. Im vorliegenden Beitrag wird die aktuelle Evidenz zu Auswirkungen des Klimawandels auf die Gesundheit beleuchtet. Zudem werden Strategien betrachtet, mit deren Hilfe der Gesundheitssektor seine Umweltfolgen reduzieren und zugleich eine qualitativ hochwertige Versorgung aufrechterhalten kann.

## Abstract (en)

The climate crisis has been identified as the largest threat to human health; paradoxically, the healthcare sector is responsible for 5% of the global greenhouse gas emissions that are driving this crisis. These emissions are largely due to carbon-intensive facilities, energy use, complex global supply chains, transportation and pharmaceuticals. In its role of safeguarding the health of both current and future populations, the healthcare sector must take actions to minimise its environmental impact. Strategies for emission reduction include sustainable infrastructure, clinical practice innovations, and procurement and supply chain reform. This article aims to examine current evidence on the health impacts of climate change and explore strategies through which the healthcare sector can reduce its environmental impact while continuing to deliver high-quality care.

## Einleitung

Der Klimawandel gilt als größte Bedrohung für die menschliche Gesundheit [1]. Die Berührungspunkte zwischen Klimawandel und öffentlicher Gesundheit sind darin ersichtlich, dass anthropogene Treibhausgas (THG)-Emissionen Umweltbedingungen verändern und sich in der Folge auf die Gesundheit des Menschen auswirken.

Der Klimawandel gilt als größte Bedrohung für die menschliche Gesundheit

Paradoxerweise hat der Gesundheitssektor wesentlichen Anteil am THG-Ausstoß: Er ist verantwortlich für etwa 5 % der weltweiten Emissionen [2]. Angesichts seines Auftrags, die Gesundheit zu schützen, hat der Sektor eine ethische Pflicht, seine negativen Auswirkungen auf die Umwelt zu minimieren.

Im vorliegenden Beitrag sollen die Folgen des Klimawandels für die Gesundheit beleuchtet werden. Zudem wird die Rolle des Gesundheitssektors in der Senkung der THG-Emissionen bei gleichzeitiger Erhaltung oder sogar Verbesserung der Patientenversorgung herausgearbeitet.

## Auswirkungen des Klimawandels auf die Gesundheit

Der Klimawandel hat wesentlichen Einfluss auf die menschliche Gesundheit.

### Direkte Effekte

Der Klimawandel trägt zu einem Anstieg von Extremwetterereignissen wie Hitzewellen, Dürren, extremen Niederschlägen und Waldbränden bei [1]. Im Jahr 2023 erlebten Säuglinge und Erwachsene über 65 Jahre eine Rekordzahl an Hitzetagen – im Durchschnitt 13,8 pro Person gegenüber 4,7 Hitzetagen im Zeitraum von 1886 bis 2005 [1]. Während Hitzewellen steigt die Gesamtmortalität an, wobei die Haupttodesursache Herz-Kreislauf-Erkrankungen sind [3]. Bei vulnerablen Personen, etwa mit chronischer Erkrankung oder extremem Alter, ist die Mortalität erhöht [3]. Eine Analyse der Hitzewelle, die Europa zwischen dem 23.06. und 02.07.2025 traf, hat ergeben, dass sich in 12 Großstädten hitzebedingt insgesamt 2305 zusätzliche Todesfälle ereigneten, wobei 60 % dem menschengemachten Klimawandel zuzuschreiben waren [4]. Hitzewellen beeinträchtigen auch unsere Wirtschaft. Im Jahr 2023 gingen schätzungsweise 512 Mrd. Arbeitsstunden durch sie verlorenen [1].

Im Jahr 2017 traf der Hurrikan Maria auf Puerto Rico, was zu einem Anstieg der Mortalität um 60 % in den darauf folgenden 3 Monaten führte [5]. Eine Notaufnahme berichtete, dass 10 % der verletzungsbedingten Vorstellungen mit dem Wirbelsturm zusammenhingen [6]. Neben dem direkten Effekt durch Verletzungen beeinträchtigten Schäden an der kritischen Infrastruktur die Gesundheit der Bevölkerung noch für Jahre, erkennbar an einem erhöhten Mortalitätsrisiko in Bezug auf Erkrankungen wie Herz-Kreislauf-Erkrankungen und Morbus Alzheimer [7].

### Indirekte Effekte

Hitzewellen sind mit weiteren Gesundheitsrisiken verbunden, da sie die Nahrungsmittelsicherheit einer Population gefährden [8]. Bedingt ist dies größtenteils durch eine Verringerung der landwirtschaftlichen Erträge und der Arbeitskraft, durch Lieferkettenunterbrechungen und eingeschränkten Zugang zu Wasser und sanitären Einrichtungen [8]. Im Zuge der Globalisierung sind diese Auswirkungen entlang der Lieferketten spürbar, am vulnerabelsten sind allerdings Bauern und indigene Völker. Indigene Kinder leiden in höherem Maße an Fehlernährung als nichtindigene Gleichaltrige, entsprechend sind sie bei Nahrungsmittelknappheit am anfälligsten [9].

Durch Vektoren übertragene Erkrankungen sind klimaempfindlich, da die Überlebensfähigkeit des Vektors vom Klima abhängig ist. Dengue ist ein Beispiel für klimasensible Erkrankungen, deren Inzidenz bereits zunimmt. Die globale Krankheitslast durch Dengue ist im Laufe der vergangenen 3 Jahrzehnte drastisch gestiegen. Im Jahr 2024 wurden über 7,6 Mio. Fälle dokumentiert [10]. Die Übertragung erfolgt über zwei Stechmückenarten der Gattung *Aedes*. Das durchschnittliche Übertragungsrisiko (R0 – Basisreproduktionszahl) hat für beide Hauptvektoren zwischen den Zeiträumen 1951–1960 und 2014–2023 zugenommen: *Aedes albopictus* (46,3 %) und *Aedes aegypti* (10,7 %), wobei der größte Anstieg in Ländern mit hohem Human Development Index (HDI) zu verzeichnen war [1].

Die rapiden Klimaveränderungen gehen mit einem rasch steigenden Ausmaß der Klimamigration einher

Im Laufe der Geschichte haben ökologische Veränderungen die Migrationsbewegungen geformt. Die rapiden Klimaveränderungen gehen mit einem rasch steigenden Ausmaß der Klimamigration einher. Das International Panel on Climate Change (IPCC) prognostiziert, dass im Jahr 2050 weltweit über 1 Mrd. Menschen küstenspezifischen Klimarisiken ausgesetzt sein könnten, was potenziell 10–100 Mio. Menschen zwingen würde, ihre Heimat zu verlassen [11].

Eine systematische Übersichtsarbeit zu den Gesundheitseffekten von Klimamigration betont als Kernprobleme eine schlechtere psychische Gesundheit, eine unsichere Versorgung mit Nahrungsmitteln und Wasser, fehlenden Wohnraum, einen Mangel an sanitären Einrichtungen sowie Marginalisierung [12]. Darüber hinaus wird die Migration aus ländlichen in vorstädtische bzw. städtische Gebiete wohl von 55 % im Jahr 2018 auf 68 % im Jahr 2050 steigen [13]. Dies geht einher mit spezifisch erhöhten Gesundheitsrisiken durch Krebs, Hypertonie, Herz-Kreislauf-Erkrankungen und Diabetes mellitus Typ 2 [12].

Durch vorausschauende Planung lassen sich Klimamigration und die Vulnerabilität von Migrantenpopulationen verringern sowie Gesundheitsrisiken reduzieren. Nach Schätzungen der Boston Consulting Group werden 2030 7 Mio. Arbeitskräfte für den notwendigen Ausbau erneuerbarer Energien fehlen [14]. Ein globales Regelwerk für Klimamigration unter Schaffung sicherer und legaler Wege für Arbeitskräfte zur Besetzung solcher offener Stellen könnte die Energiewende beschleunigen und einige Vulnerabilitäten der Migrantengemeinschaften reduzieren, beispielsweise soziale Isolation und wirtschaftliche Nachteile.

Indirekte Effekte auf die Gesundheit können weitreichend sein. Gemäß Studiendaten haben Frauen nach klimabezogenen Katastrophen ein erhöhtes Risiko, interpersonelle Gewalt zu erfahren; Frauen, die angaben, mehr als vier Katastrophen erlebt zu haben, hatten eine acht-fache Wahrscheinlichkeit [15].

## Klimawandel und gesundheitsbezogene Ungleichheiten

Die Auswirkungen des Klimawandels auf die Gesundheit sind ungleich verteilt. Sozioökonomisch benachteiligte Gruppen, indigene Gemeinschaften, ältere Menschen, Kinder und Personen mit Vorerkrankungen sind besonders anfällig. Strukturelle Ungleichheiten, beispielsweise Rassismus, ein eingeschränkter Zugang zur Gesundheitsversorgung und Umweltexpositionen, verschärfen dies noch. Zudem können Maßnahmen zur Reduktion von CO_2_, soweit sie nicht unter Berücksichtigung dieser Faktoren gestaltet und evaluiert werden, die Ungleichheiten weiter verstärken. So lässt sich die Klimakrise als Bedrohung für die öffentliche Gesundheit betrachten, gegen die systematisch vorgegangen werden muss.

## CO_2_-Fußabdruck des Gesundheitssektors

Der Gesundheitssektor ist für etwa 5 % des THG-Ausstoßes weltweit verantwortlich [2]. Innerhalb des Sektors können Emissionen drei Kategorien zugeordnet werden: Kategorie 1 – aus direkt im Besitz befindlichen/kontrollierten Quellen; Kategorie 2 – indirekte Emissionen aus eingekaufter Energie; Kategorie 3 – alle weiteren indirekten Emissionen aus Gütern, Dienstleistungen und Lieferketten [16]. Untersuchungen des CO_2_-Fußabdrucks haben die wesentlichen THG-„Hotspots“ in Gesundheitssystemen aufgedeckt: Einrichtungen und Energieverbrauch (10 % des Fußabdrucks), Emissionen durch Lieferketten und Transport (22 % weltweit) und Medikamente/Chemikalien [2, 16]. Diese Analysen sind von Bedeutung, wenn Strategien zur Emissionsreduktion entwickelt werden.

## Strategien zur Emissionsreduktion im Gesundheitswesen

Angesichts ihres erheblichen Beitrags zum menschengemachten Klimawandel müssen die Gesundheitssysteme weltweit auch ihren Beitrag zur Reduktion von Emissionen und zur Eindämmung der Klimafolgen leisten. Die Weltgesundheitsorganisation (WHO) hat die Alliance for Transformative Action on Climate and Health (ATACH) ins Leben gerufen. Von den 94 beteiligten Staaten haben sich 45 zum Ziel einer Netto-Null-Bilanz bekannt [17]. Dennoch steigen die mit dem Gesundheitssektor zusammenhängenden THG-Emissionen weiter an: im Jahr 2021 um 9,5 % gegenüber 2020 und um 36 % gegenüber 2016 [1]. Strategien zur Verminderung der Emissionen gehen häufig einher mit Kosteneinsparungen und einer verbesserten Patientenversorgung. Die Bereiche für Emissionsreduktionen können grob in drei Kategorien eingeteilt werden: i) nachhaltige Infrastruktur, ii) Innovationen in der klinischen Praxis sowie iii) Verbesserungen in Bezug auf Beschaffung und Lieferketten.

## Nachhaltige Infrastruktur

Am Gesamteinfluss der Gesundheitsversorgung auf die Umwelt hat die Infrastruktur erheblichen Anteil. Health Care Without Harm (HCWH) ist eine internationale Nichtregierungsorganisation, die weltweit mit Gesundheitsdienstleistern zusammenarbeitet, um die Umweltfolgen des Sektors zu verringern. Zusammen mit der WHO hat HCWH eine Reihe spezifischer Maßnahmen in Bezug auf Energieeffizienz, grünes Gebäudedesign, Erzeugung erneuerbarer Energien, Transport, Nahrungsmittel, Abfall und Wasser erarbeitet [18].

### Energieeffizienz

Mangelhafte Energieeffizienz trägt zu den THG-Emissionen von Krankenhäusern bei und geht zudem mit einer erheblichen finanziellen Belastung einher. Kliniken haben eine sehr hohe Energieintensität, bedingt durch ihren ununterbrochenen Betrieb. In Operationssälen müssen strikte Vorgaben zu Temperaturregelung, Luftwechselraten und kontinuierlicher Belüftung eingehalten werden, um Sterilität zu gewährleisten; dies trägt zu ihrer Energieintensität bei [19].

Vielfältige Strategien zur Verbesserung der Energieeffizienz können verfolgt werden: Durchführung von Energieaudits, Nachrüstung von Systemen mit höherer Energieeffizienz in älteren Gebäuden, Optimierung der Beleuchtung, smarte Energiemanagementsysteme und Integration erneuerbarer Energien. Einfache Maßnahmen wie das Abstellen von Heizungs‑, Lüftungs‑, Klima- und Kältesystemen können zu signifikanten Energieeinsparungen führen [20].

### Energiegewinnung

Angesichts der Energieintensität von Krankenhäusern steckt im Übergang zu 100 % erneuerbaren Energien das Potenzial, die Umweltfolgen der Gesundheitsversorgung enorm zu reduzieren. Mit der Einrichtung von Solaranlagen vor Ort kann der Energiebedarf einer Klinik zu einem Großteil gedeckt werden [21]. Nach Schätzungen sind nahezu 1 Mrd. Menschen in Ländern mit niedrigem bis mittlerem Einkommen auf Gesundheitseinrichtungen angewiesen, die über keine verlässliche Stromversorgung verfügen [22]. Durch dezentralisierte Gewinnung von Sonnenenergie könnten diese Einrichtungen mit Strom versorgt werden – ohne die Verzögerungen, die häufig mit dem Erfordernis eines Netzanschlusses einhergehen [22]. Folglich hat Sonnenenergie ein enormes Potenzial hinsichtlich der Reduktion globaler Ungleichheiten in der Gesundheitsversorgung.

Wärmepumpen werden sowohl in privat als auch in gewerblich genutzten Gebäuden zunehmend eingesetzt. Ein Krankenhaus im dänischen Sønderborg hat zwei Wärmepumpen installiert, die mithilfe von Abwärme eine wirksame Heizung und Kühlung gewährleisten; sie decken den Energiebedarf der Klinik und speisen überschüssige Wärme in das lokale Wärmenetz ein [23].

Wo die Umsetzung von Projekten vor Ort beschränkt ist, bieten „power purchase agreements“ als spezielle Form von Stromlieferverträgen Krankenhäusern die Möglichkeit, Projekte im Bereich erneuerbarer Energien zu finanzieren, die Strom ins Netz einspeisen; die Kliniken beziehen dann Strom aus dem Netz zu einem Festpreis. Modellierungen der Stiftung Possible haben ergeben, dass zwei NHS-Trusts des britischen Gesundheitssystems durch Nutzung kommunaler Windenergieprojekte, welche Gewinne von Aktionären zurück in die Kommune lenken, über einen Zeitraum von 10 Jahren 2,6 Mio. £ an Gesundheitskosten einsparen könnten [24].

Die vermehrte Einbeziehung erneuerbarer Energiequellen hat das Potenzial, Gesundheitseinrichtungen vor Energiekostenschwankungen zu schützen, sie besser gegen Stromausfälle und andere Störungen zu wappnen und den THG-Ausstoß zu senken.

### Grünes Gebäudedesign und Zertifizierung

Die Zertifizierung grüner Gebäude hat geholfen, die Aufnahme neuer Schlüsseltechnologien in grüne Gebäudedesigns zu beschleunigen. Ein breit angewendetes Rahmenwerk für nachhaltige Klinikentwürfe ist die „Leadership in Energy and Environmental Design“ (LEED)-Zertifizierung. Dabei handelt es sich um einen Katalog von Standards für ökologisches Gebäudedesign, beispielsweise zum Einsatz fortschrittlicher Belüftungssysteme. Die Umsetzung von LEED-Maßnahmen kann den Gesamtenergieverbrauch eines Gebäudes schätzungsweise um 30–50 % im Vergleich zu nichtzertifizierten Gebäuden reduzieren [25].

In Zeiten des Klimawandels ist es zunehmend erforderlich, beim Bau neuer Kliniken deren Widerstandsfähigkeit zu berücksichtigen, mit hochwasserbeständigen Bauten, hitzebeständigen Materialien und dezentralisierten Quellen erneuerbarer Energie [19].

### Nahrungsmittel

Die globale Lebensmittelproduktion ist für ein Drittel aller THG-Emissionen verantwortlich [26], wobei in Großbritannien ein großer Anteil (70 %) allein auf rotes Fleisch und Milcherzeugnisse zurückgeht [1]. Ein gesteigerter Verzehr pflanzenbasierter Nahrungsmittel ist erwiesenermaßen mit einer gesenkten Prävalenz von Erkrankungen wie Herzkrankheit, Adipositas, Diabetes mellitus Typ 2 und Krebs assoziiert, zudem mit einer Reduktion der Gesamtmortalität [27]. Im Vergleich zu einer fleischreichen Kost verursacht eine vegane Ernährung 25,1 % weniger THG-Emissionen [28].

Pflanzenbasierte Mahlzeiten in Krankenhäusern senken die Umweltfolgen der Verpflegung

Pflanzenbasierte Mahlzeiten in Krankenhäusern senken die Umweltfolgen der Verpflegung und wirken sich positiv auf die Gesundheit der Patienten aus [1, 27]. Im Jahr 2022 kooperierte die Organisation Greener By Default mit 11 Kliniken aus New York City, um Speisepläne auf Grundlage des Plant-forward-Konzepts mit überwiegend pflanzenbasierten Zutaten einzuführen, was eine erhebliche Kostenersparnis (500.000 $ jährlich) und THG-Einsparung (Reduktion des CO_2_-Fußabdrucks um ein Drittel) mit sich brachte [29].

### Abfall

Abfälle machen einen relativ kleinen Anteil des CO_2_-Fußabdrucks der Gesundheitsversorgung aus (ca. 5 % [16]), können aber bedingt durch die toxischen Eigenschaften der entsorgten Chemikalien/Materialien weitreichende Auswirkungen auf die Umwelt haben [30]. Als System sollte das Gesundheitswesen die Vermeidung von Abfällen anstreben und dabei auf eine Kreislaufwirtschaft zusteuern, in der Produkte für Wiederverwertung und Langlebigkeit ausgelegt sind. Dies verlangt grundlegende Veränderungen in Lieferketten. Diverse europaweite Initiativen haben sich zum Ziel gesetzt, das Kreislaufprinzip in Gesundheitssystemen zu stärken und die Verwendung von Einwegkunststoffen zu verringern [30]. Ein solches Projekt ist *PVC-Free Healthcare.* PVC ist ein wichtiger Werkstoff in Medizinprodukten. Seine Herstellung und Entsorgung sind mit beträchtlichen Umwelt- und Gesundheitsrisiken verbunden, bedingt durch die Freisetzung giftiger Dioxine [31]. Das Projekt versucht Alternativen zu diesem toxischen und schwer recycelbaren Stoff zu finden [31].

Bestimmte Abfallströme im Gesundheitswesen verlangen eine sichere Entsorgung, um Gesundheitsrisiken für den Menschen zu vermeiden; Beispiele sind infektiöse Abfälle, scharfe und spitze Gegenstände sowie toxische Pharmazeutika [30]. Gegenwärtig wird ein Großteil dieser Abfälle verbrannt, wobei es zu einem breiten Spektrum toxischer Emissionen kommt, unter anderem von CO_2_ und Stickoxiden [30]. Alternativen zur Verbrennung, die einen geringeren THG-Ausstoß haben, sind Autoklavieren, Recycling und Abfallvergärung [30]. Auch Mitarbeitende des Gesundheitswesens spielen eine Schlüsselrolle in der Verminderung abfallbedingter Umweltfolgen, indem sie sicherstellen, dass nur Abfälle in die emissionsreichen Abfallströme gelangen, die eine solche Beseitigung auch erfordern.

## Innovationen in der klinischen Praxis

### Telemedizin

In Großbritannien verursachen die Reisestrecken von Patienten 5 % des NHS-weiten CO_2_-Fußabdrucks [16]. Laut Schätzungen von NHS England könnten etwa 232 Mio. Fahrtmeilen (373 Mio. km) jährlich durch virtuelle Termine vermieden werden – das entspricht einer potenziellen Einsparung von 426 t CO_2_-Äquivalent (CO_2_e) pro Jahr [16]. In größeren und ländlicheren Regionen hat die Problematik eine höhere Tragweite. Eine Studie zur Situation in British Colombia, Kanada, schätzt die durchschnittliche Reisestrecke auf 18,94 km (2,89 kg CO_2_e) pro Notaufnahmebesuch in städtischen Gebieten und auf 91,2 km (13,92 kg CO_2_e) im ländlichen Raum [32]. Eine vermehrte Nutzung von virtuellen Terminen und Telegesundheit könnte diese Emissionen senken [32]. Allerdings sind telemedizinische Leistungen nicht CO_2_-neutral, da auch die eingesetzten Computersysteme und das Server-Hosting von Onlinediensten THG-Emissionen verursachen. Nach Schätzungen wäre ein Nutzen gegeben, wenn Patienten eine Reisestrecke von > 7,2 km zurücklegen müssen [33]. Die zunehmende Integration von Telemedizin könnte Ungleichheiten in der Gesundheitsversorgung verschärfen, beispielsweise durch Unterschiede im digitalen Bildungsniveau; dies unterstreicht die Bedeutung intersektionaler Analysen, um sicherzustellen, dass die grüne Transformation auch gerecht ist [34].

### Gesundheitsvorsorge

Sich auf die Prävention gesundheitlicher Beeinträchtigungen zu konzentrieren, ist von Natur aus nachhaltig, da so der Bedarf an Gesundheitsleistungen und deren Inanspruchnahme reduziert werden. Das Programm Getting It Right First Time (GRIFT; zu Deutsch: „Es beim ersten Mal hinbekommen“) hat zum Ziel, die richtige *erste *Intervention für Patienten zu identifizieren, wodurch seltener Revisionen bzw. erneute Operationen notwendig werden [35]. Ein sich abzeichnendes Thema ist, das eine frühe und intensive Rehabilitation die Aufenthaltsdauer, postoperative Komplikationen und die Kosten senken könnte – allesamt assoziiert mit erhöhten Emissionen [35]. Eine präoperative Versorgung, die sich auf Interventionen wie Anämiediagnostik, körperliche Übungen und präoperative Gehhilfen fokussiert, hat das Potenzial, Komplikationen um 30–80 % zu reduzieren [36], einhergehend mit der Einsparung von Emissionen und Kosten.

Die Nephrologie ist eines der ressourcenintensivsten Fächer im Gesundheitsbereich, insbesondere in den späteren Erkrankungsstadien, wenn eine Nierenersatztherapie erforderlich ist [37]. Hämodialyse und Peritonealdialyse gehen mit beträchtlichen Emissionen einher; diese sind bedingt durch regelmäßige Anfahrten von Patienten und Personal, Verbrauch großer Mengen Wasser und Energie sowie anfallende Kunststoffabfälle [37].

KitNewCare ist eine von der Europäischen Union (EU) finanzierte öffentlich private Partnerschaft, die evidenzbasierte und skalierbare Interventionen entwickeln soll, um Qualität und Nachhaltigkeit der nephrologischen Versorgung zu verbessern. Nach Identifizierung der Innovationen von KitNewCare werden über 450 Leistungserbringer in mehr als 8 europäischen Ländern sie umsetzen [38]. Prävention hat eine Schlüsselfunktion im Dekarbonisierungsmodell mit ihrer Zielsetzung, die Raten an frühen Diagnosen und wirksamen frühen Therapien zu steigern, um eine Progression zu verhindern [38].

### Anästhesie

Schon lange ist bekannt, dass Anästhesiegase die globale Erwärmung verstärken – und Anästhesisten und Anästhesistinnen versuchen in vorderster Reihe, die Auswirkungen abzumildern. Der NHS berichtet eine Senkung der Emissionen flüchtiger Anästhesiegase von 8000 t CO_2_e im Jahr 2018 auf 1000 t CO_2_e 2025 [39]. Zurückzuführen ist diese Entwicklung größtenteils auf die signifikant reduzierte Verwendung von Desfluran, einem flüchtigen Gas mit einem 2540-fach höheren Treibhauspotenzial als CO_2_ über einen Zeitraum von 100 Jahren [40]. Die Europäische Kommission hat ein Verbot der Verwendung erlassen, welches im Januar 2026 in Kraft tritt [41].

Des Weiteren sind in der Anästhesie stark reduzierte Emissionen von Distickstoffmonoxid (N_2_O; Lachgas) zu verzeichnen. Erreicht wurde dies hauptsächlich durch die Außerbetriebnahme von Rohrleitungsnetzen, die erwiesenermaßen für den Verlust von bis zu 99 % N_2_O durch Lecks und Gasflaschen mit überschrittenem Verfallsdatum verantwortlich sind [42].

## Verbesserungen in Bezug auf Beschaffung und Lieferketten

Nach Schätzungen sind Lieferketten für mehr als die Hälfte der mit dem Gesundheitswesen zusammenhängenden Emissionen weltweit verantwortlich [43]. Gesundheitssysteme nehmen auf diese Emissionen keinen direkten Einfluss, allerdings verfügen sie über eine beträchtliche Kaufkraft und können auf die Organisationen einwirken, die diesen Einfluss besitzen. NHS England hat viele Schritte unternommen, um Lieferanten zur Dekarbonisierung ihrer Aktivitäten zu bewegen und so bis 2030 das Ziel einer Netto-Null-Lieferkette („net zero supply chain“) zu erreichen [16]. Zu diesen Schritten zählt die Verpflichtung aller Anbieter mit Verträgen über > 5 Mio. £, einen Plan zur Reduktion von Emissionen der Kategorien 1 und 2 zu veröffentlichen; ab 2027 gilt die Verpflichtung dann für alle Verträge [44].

Komplexität, Dezentralisierung und Globalisierung von Lieferketten stellen Herausforderungen bei deren Dekarbonisierung dar. Um diese Problematik anzugehen, wurde bei der 26. UN-Klimakonferenz die Sustainable Markets Initiative Health Systems Task Force (Arbeitsgruppe zum Thema Gesundheitssysteme der Initiative für nachhaltige Märkte) eingerichtet, eine öffentlich-private Partnerschaft, in der CEO führender Pharmaunternehmen und globaler Gesundheitsdienstleister zusammenkommen [43]. Mitglieder aus dem privaten Sektor haben sich zu Zielen wie Netto-Null-Emissionen bis 2045 und 80–100 % erneuerbarer Energien im eigenen operativen Bereich bis 2030 bekannt [43].

Ein wichtiges Instrument zur Einschätzung der Umweltfolgen von Einwegartikeln ist die LZA

Ein wichtiges Instrument zur Einschätzung der Umweltfolgen von Einwegartikeln gegenüber wiederverwendbaren Produkten ist die Ökobilanz oder auch Lebenszyklusanalyse (LZA), eine Methode, mit der die Auswirkungen eines Produkts, eines Verfahrens oder einer Dienstleistung auf die Umwelt umfassend geprüft werden sollen, durchgeführt im Einklang mit internationalen Standards [45]. Mithilfe von LZA wurde eine Evidenzbasis für den Wechsel von Einweginstrumenten zu wiederverwendbaren Alternativen geschaffen, etwa zu wiederverwendbaren Laryngoskopen [46] und Vaginalspekula [47]. Die Analysen zeigen auf, wie die Hauptquellen der mit Einwegprodukten assoziierten THG-Emissionen häufig in der Rohstoffgewinnung und Entsorgung liegen (entsprechend könnte dann die Wiederverwertungs‑/Recycling-Fähigkeit einen starken Effekt haben), während bei wiederverwendbaren Produkten die Umweltauswirkungen größtenteils von der zur Wiederwertung der Produkte nötigen Energie abhängig sind und durch Nutzung erneuerbarer Energiequellen eingedämmt werden können [46].

## Schlussfolgerungen

Der Gesundheitssektor muss seinen primären Auftrag zum Schutz der Gesundheit mit der Tatsache in Einklang bringen, dass sein gegenwärtiger Betrieb negative Folgen für die Gesundheit der Menschen und den Planeten hat. Klimaschutz im Gesundheitswesen ist sowohl ein moralisches Gebot als auch eine praktische Notwendigkeit. Viele Maßnahmen zur Erreichung von Nachhaltigkeit sind kosteneffektiv *und *erhöhen die Qualität der Patientenversorgung.

Es bestehen jedoch Hindernisse. Zu den wichtigsten Herausforderungen für Gesundheitssysteme zählen finanzielle Einschränkungen, ein Fehlen regulatorischer Anreize, fragmentierte Führungsstrukturen und Widerstand gegen Veränderungen. Zur Überwindung dieser Barrieren braucht es einen koordinierten Ansatz, der wesentliche Stakeholder wie Regierungen, Nichtregierungsorganisationen, Partner aus der Industrie, Fachleute und die Öffentlichkeit einbezieht. Wichtig ist auch, dass Dekarbonisierung nicht die Patientenversorgung beeinträchtigen oder Ungleichheiten in der Gesundheitsversorgung verstärken sollte. Um dies zu vermeiden, sollten geplante Interventionen einer intersektionalen Analyse unterzogen werden, zudem sind die Langzeiteffekte von Interventionen auf die Gesundheit zu untersuchen.

Der Klimawandel ist eine der dringlichsten Bedrohungen für die menschliche Gesundheit. Durch Senkung der THG-Emissionen und Aufbau klimaresilienter Gesundheitssysteme kann das Gesundheitswesen eine Transformation gestalten, die quer durch die Gesellschaft notwendig ist.

## Fazit für die Praxis

- Der Klimawandel gilt als größte Bedrohung für die menschliche Gesundheit. Paradoxerweise hat der Gesundheitssektor wesentlichen Anteil am Treibhausgas (THG)-Ausstoß.
- Die Auswirkungen des Klimawandels auf die Gesundheit sind ungleich verteilt, dadurch wird Ungleichheit in der Gesundheitversorgung verstärkt.
- Die Infrastruktur hat einen erheblichen Anteil am Gesamteinfluss der Gesundheitsversorgung auf die Umwelt.
- Die Zertifizierung grüner Gebäude hilft, die Aufnahme neuer Schlüsseltechnologien in grüne Gebäudedesigns zu beschleunigen.
- Pflanzenbasierte Mahlzeiten in Krankenhäusern senken die Umweltfolgen der Verpflegung und wirken sich positiv auf die Gesundheit der Patienten aus.
- Sich auf die Prävention gesundheitlicher Beeinträchtigungen zu konzentrieren, ist von Natur aus nachhaltig, da so der Bedarf an Gesundheitsleistungen und deren Inanspruchnahme reduziert werden.
- Ein wichtiges Instrument zur Einschätzung der Umweltfolgen von Einwegartikeln ist die Lebenszyklusanalyse (LZA).
